# The State Board of Health Bill

**Published:** 1902-12

**Authors:** 


### TEXAS MEDICAL JOURNAL.

AUSTIN, TEXAS.

A MONTHLY JOURNAL OF MEDICINE AND SURGERY.

EDITED AND PUBLISHED BY
F. E. DANIEL, M. D.

ASSOCIATE EDITOR:
WITTEN BOOTH RUSS, M. D.,
San Antonio, Texas.

Published Monthly at Austin, Texas. Subscription price $1.00 a year in advance.

Eastern Representative: John Guy Monihan, St. Paul Building, 220 Broadway, New York City.

Official organ of the the West Texas Medical Association, the Houston District Medical Association, the Austin District Medical Society, the Brazos Valley Medical Association, the Galveston County Medical Society, and several others.

### THE STATE BOARD OF HEALTH BILL.

The committee of the State Medical Association appointed to prepare a bill for a State Board of Health have not yet agreed upon all the details of a bill, as I had hoped they would do ere now, so that the bill could appear in this issue of the JOURNAL. I hope to be able to submit it to the medical profession of the State in next issue, and to the Governor soon after his inauguration. A meeting of the committee (at Austin) has been requested for December 23rd inst., at the time of the annual meeting of the Austin District Medical Society, when it is hoped that every detail will be satisfactorily arranged.

State Health Officer Tabor has recommended in his report that a State Board of Health be created in conjunction with the Quarantine Department, and Governor Sayers has advised it. Heretofore, for many years, every effort to get a State Board of Health has been deefated because it antagonized the Quarantine Department. It will be worse than folly to make another attempt along that line, hence I deem it the wisest policy to let the Quarantine Department alone, and ask for legislation "to enlarge the scope and power of the present health system of the State for the better protection of the public health; to create a State Board of Health and Vital Statistics in conjunction with the Quarantine Department." etc. No appropriation will be asked for, as the revenues from the Quarantine Department, it is believed, will be sufficient to meet the expenses of the proposed board. More anon.

